# Bio-Organic Fertilizer Modulates the Rhizosphere Microbiome to Enhance Sugarcane Growth and Suppress Smut Disease

**DOI:** 10.3390/microorganisms13112563

**Published:** 2025-11-10

**Authors:** Fei Chen, Xunyang He, Qiumei Liu, Fulai Gao, Chaozhen Zeng, Dejun Li

**Affiliations:** 1 College of Life Science and Technology, Central South University of Forestry and Technology, Changsha 410004, China; 17308530078@163.com; 2 Institute of Subtropical Agriculture, Chinese Academy of Sciences, Changsha 410125, China; hbhpjhn@isa.ac.cn (X.H.); liuqiumei@isa.ac.cn (Q.L.); 17638256904@163.com (F.G.); 3 Guangxi Key Laboratory of Karst Ecological Processes and Services, Huanjiang Observation and Research Station for Karst Ecosystems, Chinese Academy of Sciences, Huanjiang 547100, China

**Keywords:** sugarcane smut, bio-organic fertilizer, rhizosphere microbial community, *Sporisorium scitamineum*, disease suppression, plant growth promotion

## Abstract

Sugarcane smut, caused by the fungal pathogen *Sporisorium scitamineum*, leads to significant economic losses in the global sugarcane industry. Bio-organic fertilizers (BF) offer a promising and sustainable strategy to mitigate smut incidence and enhance sugarcane growth. While the application of BF is known to modulate root exudates and rhizosphere microbial community structure, thereby promoting disease resistance, the precise mechanisms underpinning BF-mediated suppression of sugarcane smut remain largely unclear. This study investigated the microbiological mechanisms of smut suppression using a pot experiment, comparing a novel BF treatment (composted substrate enriched with *Bacillus subtilis*, *Bacillus altitudinis*, *Bacillus cereus*, *Trichoderma harzianum*, and *Trichoderma longibrachiatum*, biochar, and calcium carbonate) with a control receiving only conventional organic fertilizer. BF application significantly increased plant height (by 95.2%), dry weight (137.5%), fresh weight (253.3%), and sugar content (43.1%) relative to the control. Furthermore, the BF treatment enhanced catalase activity by 167.8% and peroxidase activity by 102.3% in sugarcane leaves, while the control effectiveness against the incidence of smut disease reached 88.0%. Analysis of the rhizosphere microbiome revealed that BF application significantly altered microbial alpha- and beta-diversity. Specifically, the BF treatment notably enriched beneficial genera such as *Pseudomonas* and *Meyerozyma*. Beta-diversity analysis revealed distinct microbial community structures in BF-treated rhizosphere soil compared to the control. Correlation and random forest analyses identified *Pseudomonas* and *Meyerozyma* as key taxa that were positively associated with sugarcane growth parameters and negatively correlated with smut incidence. These findings elucidate the dual role of this novel BF in enhancing sugarcane growth and suppressing smut incidence through the strategic reshaping of the rhizosphere microbiome.

## 1. Introduction

Sugarcane (*Saccharum officinarum* L.) is a globally vital crop for sugar and bioenergy production [1]. Cultivated in over 100 countries across 26 million hectares, it supplies approximately 80% of the world’s sugar and 40% of its bioethanol, with a total value exceeding $80 billion [1]. Sugarcane smut, caused by *Sporisorium scitamineum*, is one of the most destructive diseases affecting sugarcane production worldwide, causing substantial losses in both yield and quality [2]. The typical symptom of this disease is the emergence of a black or gray, whip-like structure (the sorus) at the plant apex following infection [3], ultimately leading to substantial yield reduction and plant death [4]. Effective prevention and control of sugarcane smut are hence imperative for the sustainability of the sugarcane industry.

Current management strategies for controlling sugarcane smut include the use of resistant varieties, hot water treatment of seed cane, the removal of diseased plants, and fungicide application [5,6]. For decades, chemical pesticides have been the primary method for protecting plants against soil-borne pathogens. However, their extensive use can lead to environmental contamination, harm to non-target organisms, the development of pathogen resistance, and the depletion of beneficial soil microflora [7]. Consequently, biological control approaches centered on microbial inoculants have emerged as a sustainable alternative [8]. Soil harbors a diverse array of microorganisms involved in nutrient cycling, organic matter transformation, and soil habitat modification, with some mediating local impacts on microbiome assembly by altering soil properties [9]. Beneficial microbial strains, including *Trichoderma*, *Bacillus*, and *Pseudomonas*, can improve plant hormonal regulation, soil health, nutrient availability and uptake, and overall plant growth [10], mitigating the adverse effects of environmental stressors [11]. While beneficial microorganisms are present in soil, their colonization and efficacy in the plant root zone are often limited [12]. Rhizosphere microorganisms, however, significantly influence plant nutrient status and facilitate the uptake of specific trace elements [8]. Strategies such as plant breeding and agricultural management practices can reshape rhizosphere microbial communities to favor plant health [13].

Bio-organic fertilizers (BF), which combine organic matter with beneficial microorganisms, represent an innovative approach to enhancing soil fertility and plant health [14]. They maintain soil richness in macro- and micronutrients and can be applied in solid, liquid, or encapsulated forms [14,15]. Bio-organic fertilizers incorporating antagonistic microorganisms can simultaneously enhance plant nutrition while suppressing soil-borne pathogens, leading to a significant reduction in disease incidence [16]. Numerous studies have demonstrated that BF application improves the colonization ability of beneficial microbes and enhances plant-growth-promoting effects, especially when combined with soil amendments [16]. BF can also improve soil physical and chemical properties, modulate microbial community structure, increase soil microbial diversity, and reduce plant disease incidence [17]. Biochar, a carbon-rich material derived from pyrolysis, has diverse applications, including as a soil amendment [18]. It creates favorable conditions for root development and microbial function by enhancing soil pH, porosity, and water availability [19]. Biochar can catalyze biotic and abiotic reactions, particularly in the rhizosphere, thereby increasing nutrient supply and uptake, reducing phytotoxins, stimulating plant growth, and enhancing resilience to diseases and environmental stressors [19]. Calcium carbonate (CaCO_3_) is an effective soil amendment for regulating soil pH, improving soil structure, and enhancing microbial biodiversity [20]. Previous research has shown BF to be effective in controlling *Fusarium* wilt in melon (*Cucumis melo*) and ginger (*Zingiber officinale*) rhizome rot [21,22]. Pathogen suppression by beneficial microbes can occur via direct antagonism (e.g., parasitism, competition for resources, production of antimicrobials, or disruption of quorum sensing) and indirect suppression through the induction of systemic resistance in the host plant [23]. Despite these advances, the specific mechanisms by which BF controls sugarcane smut disease remain largely unexplored, and novel mechanisms distinct from those in other crops may exist.

To address this knowledge gap, the present study aimed to elucidate the microbiological mechanisms underlying the suppression of sugarcane smut by a BF formulated with biochar and calcium carbonate. A controlled pot experiment was conducted using a substrate derived from cow dung and mulberry branches. Treatments included the BF supplemented with a consortium of beneficial microorganisms (*Bacillus subtilis*, *Bacillus altitudinis*, *Bacillus cereus*, *Trichoderma harzianum*, and *Trichoderma longibrachiatum*) and a conventional organic fertilizer serving as the control. We hypothesized that the BF would not only enhance sugarcane growth but also reduce smut incidence by reshaping the rhizosphere microbial community to favor beneficial, disease-suppressive taxa. The primary objectives of this study were to: (i) assess the impact of the BF on sugarcane growth and smut disease incidence, and (ii) characterize the associated shifts in rhizosphere microbial community structure. By integrating plant physiological assessments with high-throughput microbial community analyses, this research aims to provide comprehensive insights into the dual role of BF in augmenting crop productivity and enhancing disease resistance. While BFs have been studied, this research provides novel insights by (1) testing a specific formulation combining a Bacillus/Trichoderma SynCom with biochar/CaCO_3_ amendments and (2) linking the potent in situ enrichment of specific beneficial taxa, particularly the yeast *Meyerozyma*, to the successful suppression of sugarcane smut, ultimately contributing to the advancement of sustainable agricultural practices.

## 2. Materials and Methods

### 2.1. Fertilizer Preparation

The base organic fertilizer was produced by composting cow manure and powdered mulberry branches. The materials were composted with the carbon-to-nitrogen (C:N) ratio adjusted to approximately 25–30:1 and a moisture content of around 65%. Each composting pile weighed 1000 kg (dry weight) with cattle manure and mulberry branches mixed at a ratio of 8:2 (*w*/*w*, dry weight basis), and was turned every three days during the composting period. Details of similar composting processes are provided in our previous studies [24,25,26]. Beneficial microbial strains (*Bacillus subtilis*, *Bacillus altitudinis*, *Bacillus cereus*, *Trichoderma harzianum*, and *Trichoderma longibrachiatum*), previously isolated from the rhizosphere of healthy sugarcane plants in fields affected by smut disease and screened in our laboratory, were used. Bacterial strains were cultured in Lysogeny Broth (LB) and fungal strains in Potato Dextrose Broth (PDB) medium at 37 °C (bacteria) or 28 °C (fungi) with shaking at 180 rpm [27,28].The bio-organic fertilizer (BF) was produced as follows: 6% (*w*/*w*) calcium carbonate powder and 6% (*w*/*w*) biochar were added to the mature compost. The calcium carbonate powder was collected from a local quarry of limestone and sieved through a 100-mesh sieve. The biochar was produced from maize straw by pyrolyzing at 500–600 °C, and sieved to particles of 2–5 mm [25]. Bacterial and fungal strains were cultured in their respective broths until reaching the stationary phase. The cultures were then combined without centrifugation to create a mixed-species liquid inoculum. This beneficial microbial inoculum (approximately 5 L per 50 kg batch of the compost-amendment mixture), with a final concentration of approximately 1.0 × 10^8^ CFU mL^−1^ for each strain, was then mixed uniformly into the compost-amendment mixture. For each 50 kg batch of this mixture, the moisture content was adjusted to 65%, and a secondary fermentation was carried out at a constant temperature of 37 °C for approximately 15 days, with turning every two days. The control organic fertilizer was the base organic fertilizer, i.e., the same mature compost without the addition of microbial inoculants, biochar, or calcium carbonate.

The selection of these five microbial strains to form a synthetic community (SynCom) was based on their well-documented and complementary mechanisms for plant growth promotion and disease suppression. *Bacillus* species are renowned for their capacity to produce a wide array of antimicrobial lipopeptides, such as iturins and fengycins, which exhibit potent antifungal activity against a broad spectrum of plant pathogens [29]. Furthermore, they are known to induce systemic resistance in host plants, priming them for a more rapid and robust defense response upon pathogen attack [30]. *Trichoderma* species, particularly *T. harzianum*, are aggressive mycoparasites that can directly attack and degrade the cell walls of pathogenic fungi through the secretion of lytic enzymes like chitinases and glucanases [31]. They also compete effectively for nutrients and space in the rhizosphere. By combining bacteria and fungi with distinct and synergistic modes of action, namely, antibiosis and induced systemic resistance from *Bacillus* and mycoparasitism and competition from *Trichoderma*, the formulated SynCom was designed to provide a multi-pronged, robust, and resilient biocontrol effect against *S. scitamineum* while simultaneously promoting sugarcane growth [32]. The robust spore-forming nature of *Bacillus* and the resilient chlamydospores of *Trichoderma* also ensure their stability and viability in a fertilizer formulation.

### 2.2. Experimental Design

A pot experiment was conducted at the Huanjiang Observation and Research Station for Karst Ecosystems, Chinese Academy of Sciences, Huanjiang County, Guangxi Zhuang Autonomous Region, China. The sugarcane variety used was ‘Guitang 44’, a moderately smut-susceptible cultivar. Healthy seed canes were propagated from stalks collected from the field. The soil for the pot experiment was collected from a local sugarcane field, air-dried, crushed, and sieved through a 2 mm mesh to remove stones and plant debris. Two treatments were established: (i) control (base organic fertilizer) and (ii) BF (bio-organic fertilizer). Each pot contained 20 kg of soil and 1.5 kg of the respective organic fertilizer, which was thoroughly pre-mixed with the soil. To ensure equal nutrient input, NPK nutrient levels in each pot were balanced across treatments using compound fertilizer (N:P:K = 15:15:15), urea, calcium superphosphate, and potassium chloride as needed. Sugarcane seed stalks were planted 10 cm below the soil surface on 27 April 2023. After seedling emergence, all plants were inoculated by drenching the root zone with a teliospore suspension of *Sporisorium scitamineum* (1 × 10^6^ spores/mL, 50 mL per plant). Each treatment comprised 30 replicates (pots), with one plant per pot. No chemical pesticides were applied during the sugarcane growth period.

### 2.3. Disease Assessment and Sample Collection

Two months after planting, weekly assessments were conducted to count the number of diseased plants. Pots were arranged in a randomized complete block design. Sugarcane smut incidence was determined by observing characteristic symptoms (slender stems, abnormal growth at the apex, and the emergence of black, whip-like sori) and calculated as the proportion of infected plants at the end of the experiment (approximately 8 months after planting) when disease symptoms stabilized. Disease incidence (DI, %) and disease control efficacy (DCE, %) for each treatment were calculated at the end of the observation period as follows:

(1) $$DI=\frac{Number\;of\;diseased\;plants}{Total\;number\;of\;plants}\times 100$$

(2) $$DCE=\frac{DI\;in\;control-DI\;in\;treatment}{DI\;in\;control}\times 100$$

At the end of the pot experiment, sugarcane plants were harvested. Plant height, stem diameter, fresh weight, and dry weight (after oven-drying at 70 °C to constant weight) were recorded (n = 30). Sugar content was determined using a PAL-GrapeMust (Brix) refractometer (Atago Co., Ltd., Tokyo, Japan) [33]. Rhizosphere soil, defined as soil that adhered to roots after gentle shaking [34], was collected from 5 randomly selected plants per treatment for microbial analysis. Bulk soil samples were collected from the same 5 replicates for the determination of soil physicochemical properties. Leaf samples from the same plants were collected for enzyme activity analysis.

### 2.4. Determination of Enzyme Activities

Catalase (CAT) and Peroxidase (POD) activities (n = 5) in fresh leaf samples were determined using spectrophotometric methods. For POD activity, the method was adapted from Maehly and Chance (1954) [35] using guaiacol as a substrate. The reaction mixture contained 0.1 M phosphate buffer (pH 6.0), 20 mM guaiacol, 40 mM H_2_O_2_, and leaf enzyme extract. The change in absorbance at 470 nm was recorded for 3 min. One unit of POD activity was defined as the amount of enzyme causing a change of 0.005 in absorbance per minute per gram of fresh tissue (FW). POD activity (U g^−1^ FW) was calculated using Equation (3):

(3) $$POD\;activity=\frac{\Delta A470\times V_{total}}{0.005\times W\times V_{sample}\times T}$$

where ΔA470 is the difference between the absorbance at 470 nm measured at 90 s (A_2_) and at 30 s (A_1_), calculated as ΔA470 = A_2_ − A_1_; Vtotal is the total volume of the reaction system (mL); Vsample is the volume of crude enzyme solution added (mL); W is the fresh weight of the sample (g); and T is the reaction time (min).

CAT activity was measured using the ammonium molybdate colorimetric method based on the reaction of H_2_O_2_ with ammonium molybdate [36]. The reaction mixture contained 0.1 M phosphate buffer (pH 7.0), 10 mM H_2_O_2_, and leaf enzyme extract. The residual H_2_O_2_ was measured at 405 nm after reaction with ammonium molybdate. One unit of CAT activity was defined as the amount of enzyme that decomposes 1 µmol of H_2_O_2_ per minute per gram of fresh tissue (FW). CAT activity (U g^−1^ FW) was calculated using Equation (4):

(4) $$CAT\;activity=\frac{x\times V_{S2}\times V_{sampletotals}}{W\times V_{sample}\times T}$$

where x is the H_2_O_2_ concentration degraded (µmol/mL) calculated from a standard curve; VS_2_ is the volume of Reagent II added to the reaction system (mL); V_sampletotals_ is the total volume of crude enzyme solution (mL); V_sample_ is the volume of crude enzyme solution added to the reaction system (mL); W is the fresh weight of the sample (g); and T is the reaction time (min).

### 2.5. Measurements of Soil Physicochemical Properties

Bulk soil samples (n = 5) were collected for the determination of soil physicochemical properties using standard protocols [37]. Fresh soil was sieved through a 2 mm mesh to remove plant residues and then air-dried in the laboratory for subsequent analysis. Soil pH was measured in a 1:2.5 (*w*/*v*) soil-to-water suspension using a calibrated pH meter (FE20K, Mettler-Toledo, Greifensee, Switzerland). Soil organic carbon (SOC) was determined using the Walkley–Black wet oxidation method with potassium dichromate and concentrated sulfuric acid, followed by titration with ferrous sulfate. Soil total nitrogen (TN) was measured by the Dumas combustion method using an elemental analyzer (Vario MAX cube, Elementar, Langenselbold, Germany) after soil samples were finely ground to pass through a 0.15 mm sieve. Soil total phosphorus (TP) and total potassium (TK) were determined by tri-acid digestion. TP concentration was analyzed colorimetrically using the ascorbic acid molybdate method, and TK concentration was measured using an inductively coupled plasma emission spectrometer (5110 ICP-OES, Agilent, Santa Clara, CA, USA). Available nitrogen (AN) was determined by the alkaline hydrolysis diffusion method. Available phosphorus (AP) was extracted using the Olsen method with 0.5 M sodium bicarbonate (NaHCO_3_, pH 8.5) and determined colorimetrically by the molybdenum blue method. Available potassium (AK) was extracted with 1.0 M neutral ammonium acetate at a 1:10 soil-to-solution ratio and measured by ICP-OES.

### 2.6. DNA Extraction and High-Throughput Sequencing

Total DNA was extracted from 0.5 g of rhizosphere soil (n = 3 here, since two samples were contaminated during DNA extraction) using the E.Z.N.A.^®^ Soil DNA Kit (Omega Bio-tek, Norcross, GA, USA) according to the manufacturer’s instructions. The V4 region of the bacterial 16S rRNA gene was amplified using primers 515F (5′-GTGCCAGCMGCCGCGGTAA-3′) and 806R (5′-GGACTACHVGGGTWTCTAAT-3′). The ITS1 region of the fungal ITS gene was amplified using primers ITS1F (5′-CTTGGTCATTTAGAGGAAGTAA-3′) and ITS2R (5′-GCTGCGTTCTTCATCGATGC-3′). PCR reactions were performed in a 30 µL mixture containing 15 µL of Phusion^®^ High-Fidelity PCR Master Mix (New England Biolabs, Ipswich, MA, USA), 0.2 µM of each primer, and 10 ng of template DNA. Thermal cycling conditions were: initial denaturation at 98 °C for 1 min; followed by 30 cycles of 98 °C for 10 s, 50 °C for 30 s, and 72 °C for 30 s; and a final extension at 72 °C for 5 min. PCR products were purified and sequenced on an Illumina NovaSeq 6000 platform (PE250).

Raw sequences were processed using QIIME2 (Version QIIME2-202202) [38]. Briefly, sequences were demultiplexed, quality-filtered, and denoised into Amplicon Sequence Variants (ASVs) using the DADA2 plugin. Taxonomic classification was performed against the SILVA database (v138) for bacteria and the UNITE database (v8.3) for fungi. The raw 16S rRNA sequencing data and ITS sequencing data have been deposited in the China National Center for Bioinformation under the accession number CRA030229 (https://ngdc.cncb.ac.cn/gsa).

### 2.7. Statistical Analysis

Data on plant growth, disease incidence, enzyme activities, and soil properties were subjected to an independent samples *t*-test using the rstatix package in R (v4.4.0) to compare means between the two treatments. Alpha diversity indices (Shannon, Simpson’s index (1-D), Chao1, Pielou’s evenness) and beta diversity (Bray–Curtis dissimilarity) were calculated using the vegan package (version 2.6–10). Principal Component Analysis (PCA) was used to visualize differences in microbial community structure. Functional prediction of fungal communities was performed using FUNGuild (version 1.0) [39], and bacterial functional profiles were predicted using PICRUSt2 (version 2.5.2) [40] based on KEGG pathways. Correlations between the top 50 most abundant microbial genera and plant parameters or disease incidence were assessed using Spearman’s rank correlation. Random forest analysis was performed to identify key microbial taxa contributing to plant growth and disease incidence using the rfPermute package in R (v4.4.0). Significance was set at *p* < 0.05 unless otherwise stated.

## 3. Results

### 3.1. Effects of Bio-Organic Fertilizer Application on Sugarcane Growth, Disease Incidence and Enzyme Activities

**Figure 1:**
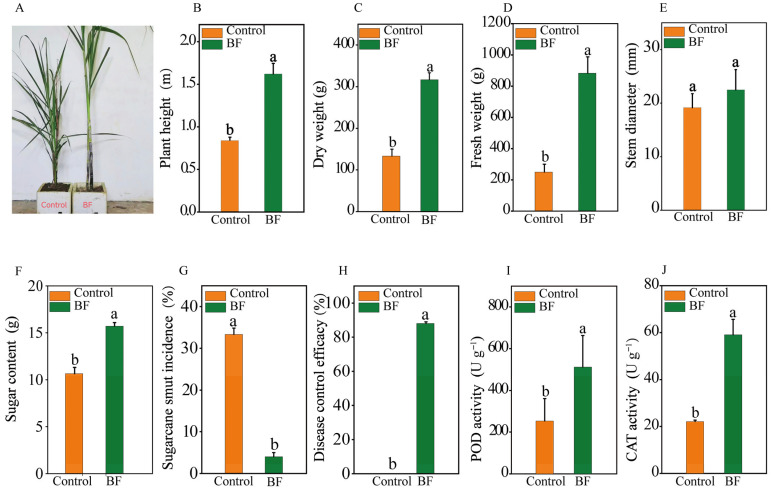
Effects of bio-organic fertilizer (BF) on sugarcane growth, sugarcane smut disease incidence, and enzyme activities. (**A**) Comparison of plant growth under different treatments; (**B**) Plant height (m); (**C**) Dry weight (g); (**D**) Fresh weight (g); (**E**) Stem diameter (mm); (**F**) Sugar content (%); (**G**) Sugarcane smut incidence (%); (**H**) Disease control efficacy (%); (**I**) Peroxidase (POD) activity (U g^−1^ FW); (**J**) Catalase (CAT) activity (U g^−1^ FW); Different letters indicate significant differences between the control and BF treatments at (independent samples *t*-test, *p* < 0.05). Data are presented as means ± standard errors (n = 30 for growth/disease parameters, n = 5 for enzyme assays).

Application of BF significantly improved sugarcane growth parameters compared to the control (Figure 1A). Plant height in the BF group (1.6 ± 0.1 m) was 95.2% greater than in the control (0.8 ± 0.0 m) (Figure 1B; *p* < 0.05). Dry weight (BF: 316.7 ± 16.7 g; Control: 133.3 ± 16.7 g) and fresh weight (BF: 883.3 ± 60.1 g; Control: 250.0 ± 28.7 g) were significantly increased by 137.5% and 253.3%, respectively, in the BF treatment (Figure 1C,D; *p* < 0.05). Nevertheless, stem diameter was comparable between the two treatments (Figure 1E; *p* > 0.05). Sugar content was 43.1% higher in BF-treated plants compared to the control (Figure 1F; *p* < 0.05). The BF application significantly reduced sugarcane smut incidence. The disease incidence in the BF treatment was 4.0 ± 0.58%, a substantial reduction from the 33.3 ± 0.88% incidence observed in the control (Figure 1G; *p* < 0.05). This represented a disease control efficacy of 88.0% for the BF treatment (Figure 1H). BF treatment significantly enhanced the activities of antioxidant enzymes in sugarcane leaves. POD activity in BF-treated plants (512.5 ± 67.2 U g^−1^ FW) was 102.3% higher than in the control (253.3 ± 48.0 U g^−1^ FW) (Figure 1I; *p* < 0.05). Similarly, CAT activity was 167.8% higher in the BF group (59.0 ± 3.8 U g^−1^ FW) compared to the control (22.1 ± 0.4 U g^−1^ FW) (Figure 1J; *p* < 0.05).

### 3.2. Effects of Bio-Organic Fertilizer Application on Soil Physicochemical Properties

The application of BF significantly altered key soil physicochemical properties compared to the control treatment (Table 1). Specifically, soil pH was significantly higher in the BF treatment (6.99 ± 0.04) than in the control (6.82 ± 0.04). The concentrations of SOC, TN, TP, AN and AP were not significantly different. Available potassium (AK) was significantly and substantially higher (2130.7 ± 60.3 mg/kg) in the BF treatment, more than double the level in the control (880.5 ± 10.1 mg/kg). Total K (TK) was numerically lower in the BF group, though not statistically significant.

### 3.3. Effects of Bio-Organic Fertilizer Application on Rhizosphere Microbial Community Composition and Diversity

**Figure 2:**
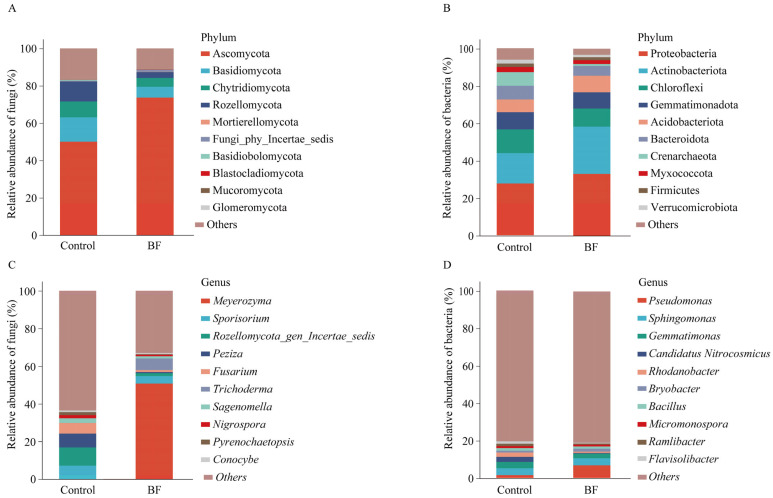
Impact of bio-organic fertilizer (BF) on rhizosphere microbial composition at phylum and genus levels (n = 3). (**A**) Relative abundance of fungal phyla; (**B**) Relative abundance of bacterial phyla; (**C**) Relative abundance of fungal genera; (**D**) Relative abundance of bacterial genera. The top 10 most abundant taxa in each category are shown, with others representing the sum of remaining taxa.

**Figure 3:**
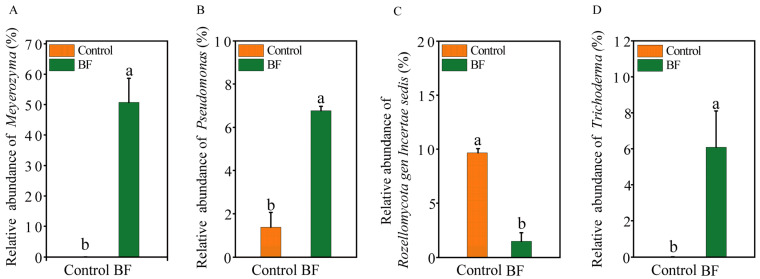
Differential abundance of key microbial genera in response to bio-organic fertilizer (BF) application (n = 3). Relative abundances of (**A**) *Meyerozyma*; (**B**) *Pseudomonas*; (**C**) *Rozellomycota* gen. incertae sedis; (**D**) *Trichoderma*. Different letters indicate significant differences between the control and BF treatments (independent samples *t*-test, *p* < 0.05). Data are presented as means ± standard errors.

At the phylum level of fungi, Ascomycota (50–74%) was the most dominant fungal phylum across all samples, followed by Basidiomycota (6–13%) (Figure 2A). For bacteria, Proteobacteria was the most dominant phylum (28–33%), followed by Actinobacteriota and Chloroflexi (9–12%) (Figure 2B). At the genus level, significant differences in the relative abundance of several key genera were observed (Figure 3A–D). For the fungal community, *Meyerozyma* was notably enriched in the BF treatment, with a relative abundance of 50.8 ± 8.0%, significantly higher than in the control (Figure 2C and Figure 3A; *p* < 0.05). *Trichoderma* also showed significantly increased relative abundance (BF: 6.09 ± 2.01%; Control: 0.02 ± 0.00%) under BF treatment (Figure 3D; *p* < 0.05). Conversely, the relative abundance of *Rozellomycota* gen. *incertae sedis* was significantly lower under BF treatment (Figure 3C; *p* < 0.05). In the bacterial community, *Pseudomonas* was significantly enriched in the BF treatment, with a relative abundance of approximately 6.8%, markedly higher than in the control (Figure 2D and Figure 3B; *p* < 0.05).

**Figure 4:**
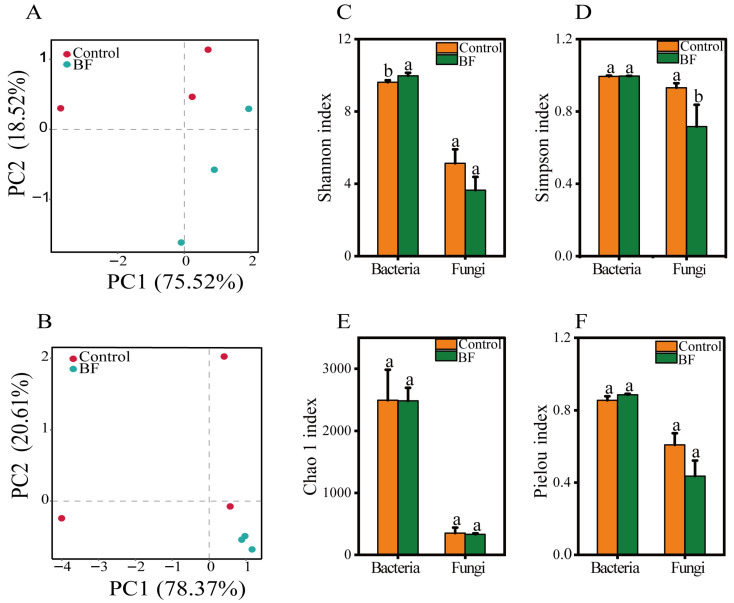
Alpha and beta diversity of rhizosphere microbial communities under the control and bio-organic fertilizer (BF) treatments (n = 3). (**A**) Principal component analysis (PCA) of bacterial communities based on Bray–Curtis dissimilarity, with PC1 and PC2 explaining 75.52% and 18.52% of total variation, respectively; (**B**) PCA of fungal communities, with PC1 and PC2 explaining 78.37% and 20.61% of variation, respectively; (**C**) Alpha-diversity indices (Shannon) for bacterial and fungal communities; (**D**) Alpha-diversity indices (Simpson) for bacterial and fungal communities. (**E**) Alpha-diversity indices (Chao 1) for bacterial and fungal communities; (**F**) Alpha-diversity indices (Pielou) for bacterial and fungal communities. Different letters indicate significant differences between the control and BF treatments at *p* < 0.05. Data are presented as means ± standard errors.

Beta-diversity analysis using PCA revealed clear distinctions in both bacterial and fungal community structures between the BF and control treatments (Figure 4A,B). For bacteria, PC1 and PC2 explained 75.5% and 18.5% of the total variation, respectively, with distinct clustering between BF and control along PC1 (Figure 4A). Similarly, for fungi, PC1 (78.4%) and PC2 (20.6%) separated the BF and control groups, indicating a significant alteration in microbial community composition due to BF application (Figure 4B).

Alpha-diversity analysis showed significant effects of BF application (Figure 4C–F). For bacteria, the Shannon index was significantly higher in the BF group compared to the control (*p* < 0.05), suggesting increased bacterial richness and evenness. For fungi, the Simpson’s index of diversity was significantly lower in the BF group (*p* < 0.05), indicating that the fungal community exhibited lower diversity, largely due to the strong dominance of the genus *Meyerozyma*. The Shannon index for fungi (Figure 4C), the Simpson index for bacteria (Figure 4D), the Chao 1 index (Figure 4E), or the Pielou index (Figure 4F) did not show significant differences between the two treatments.

### 3.4. Functional Prediction of Rhizosphere Microbiota

**Figure 5:**
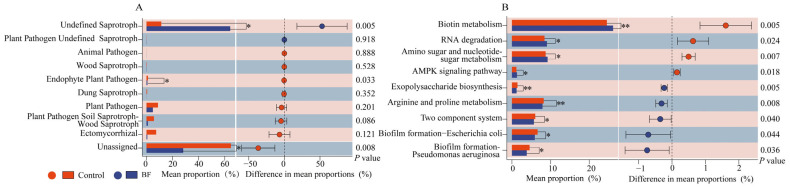
Predicted functional profiling of rhizosphere microbiota (n = 3). (**A**) Functional prediction of fungal communities via FUNGuild; (**B**) Functional prediction of bacterial communities via PICRUSt2 based on KEGG pathways. * and ** denote significant difference between control and BF at *p* < 0.05 and *p* < 0.01, respectively.

FUNGuild analysis predicted the functional roles of fungal communities (Figure 5A). The relative abundance of fungi classified as ‘Endophyte-Plant Pathogen’ was significantly reduced in the BF treatment compared to the control (*p* < 0.05). While other guilds such as ‘Undefined Saprotroph’ and ‘Unassigned’, also showed large shifts, their lack of specific functional annotation limits interpretation.

KEGG pathway analysis based on PICRUSt2 prediction for bacterial communities revealed significant changes in several metabolic pathways (Figure 5B). Functions related to Biotin metabolism, RNA degradation, Amino sugar and nucleotide sugar metabolism, and AMPK signaling pathway were significantly upregulated in the BF treatment (*p* < 0.05). Conversely, functions associated with Exopolysaccharide biosynthesis, Arginine and proline metabolism, Two-component system, Biofilm formation—*Escherichia coli* and Biofilm formation (*Escherichia coli* and *Pseudomonas aeruginosa*) were significantly downregulated (*p* < 0.05).

### 3.5. Correlations Between Rhizosphere Microbiota, Plant Growth, and Disease Suppression

**Figure 6:**
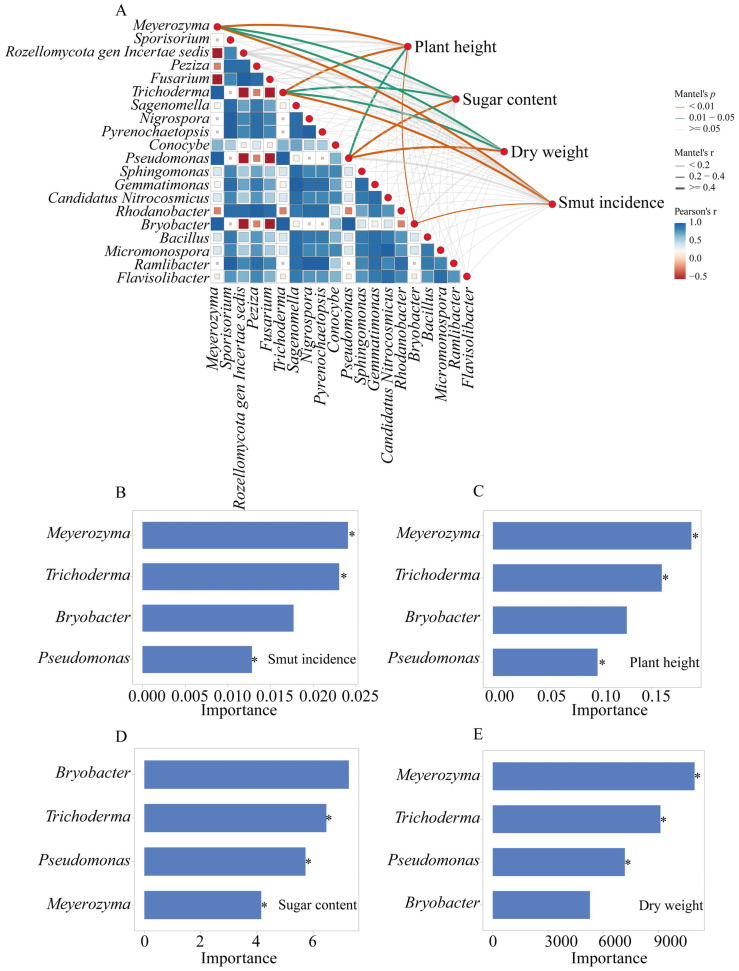
Random forest analysis of microbial genera contributing to sugarcane growth and sugarcane smut disease suppression (n = 3). (**A**) Spearman correlation analysis between relative abundances of microbial genera and sugarcane phenotypes with the size of squares denoting the degree of correlation; (**B**–**E**) Importance of the influence of key microbial genera; (**B**) sugarcane smut incidence; (**C**) plant height; (**D**) sugar content, and (**E**) dry weight. * *p* < 0.05.

Spearman correlation analysis revealed that the fungal genera *Meyerozyma* and *Trichoderma*, and the bacterial genera *Pseudomonas* and *Bryobacter* were significantly correlated with plant growth parameters (plant height, sugar content, dry weight), and significantly correlated with the incidence of sugarcane smut (Figure 6A). Random forest analysis identified the microbial genera that most significantly contributed to the observed plant responses (Figure 6B–E). *Meyerozyma*, *Trichoderma* and *Pseudomonas* were among the top contributors to the variation in smut incidence, with their higher abundance associated with lower disease levels (Figure 6B). These same genera were also identified as important contributors to plant height (Figure 6C), sugar content (Figure 6D), and dry weight (Figure 6E).

## 4. Discussion

This study demonstrates that the application of a bio-organic fertilizer formulated with beneficial microbes, biochar, and calcium carbonate significantly enhances sugarcane growth, reduces smut disease incidence, and modulates the rhizosphere microbial community structure and function. These findings provide mechanistic insights into the complex interactions between BF, soil microbiota, and plant health in the sugarcane agroecosystem.

### 4.1. Bio-Organic Fertilizer Enhances Sugarcane Growth and Induces Systemic Resistance

The observed improvements in plant height, biomass, and sugar content (Figure 1) are consistent with the growth-promoting effects of bio-organic fertilizers [41,42]. These effects can be attributed to multiple synergistic factors. First, the BF application resulted in a significant reduction in sugarcane smut incidence (88.0% control efficacy) (Figure 1G,H). This aligns with research showing that combining biocontrol agents with organic fertilizers can effectively suppress plant diseases [16,43]. This protection is likely conferred through a combination of direct antagonism by the inoculated strains (*Trichoderma* spp.) and the recruited beneficial microbes (*Pseudomonas*, *Meyerozyma*), as well as indirect mechanisms. The increased activities of antioxidant enzymes (POD and CAT) in BF-treated plants (Figure 1I,J) suggest an enhancement of the plant’s defense system. Elevated POD and CAT activities are hallmarks of the plant defense response, improving the plant’s capacity to mitigate the oxidative stress associated with pathogen attack [35,44]. This suggests that the BF primes the plant’s innate immune system for a more robust defense, a classic indicator of induced systemic resistance [44,45]. Second, the application of BF altered the soil’s physicochemical properties (Table 1). The inclusion of calcium carbonate successfully raised the soil pH, creating a more favorable environment for nutrient availability and microbial activity. Third, the BF treatment more than doubled the concentration of available K. Potassium is a crucial macronutrient for plant health, known to activate enzymes, regulate stomatal function and water balance, and enhance resilience to both biotic and abiotic stresses [46]. This elevation in available K is a likely contributor to the enhanced plant growth and physiological status.

### 4.2. Bio-Organic Fertilizer Modulates Rhizosphere Microbial Community Structure and Diversity

The application of BF led to significant shifts in the composition and diversity of the rhizosphere microbial community. The enrichment of *Pseudomonas* and *Meyerozyma* in the BF-treated rhizosphere was particularly noteworthy (Figure 3). *Pseudomonas* species are well-known plant growth-promoting rhizobacteria that can enhance plant health through multiple mechanisms, including nutrient solubilization, phytohormone production, and direct antagonism against pathogens [47,48,49]. The increased abundance of *Pseudomonas* in our study is consistent with Tao et al. [50], who reported that BF stimulated indigenous soil *Pseudomonas* populations, leading to enhanced disease suppression. A substantial increase in the abundance of the yeast *Meyerozyma* (from <0.1% to >50%) was a major driver of the community shift. *Meyerozyma* has been recognized for its biocontrol potential against various plant pathogens, often by outcompeting them for nutrients and space [51,52]. The substantial increase in *Meyerozyma* abundance in the BF treatment suggests it may be a highly effective competitor in the sugarcane rhizosphere, thereby excluding or suppressing *S. scitamineum*. The significant enrichment of *Trichoderma* (Figure 3D), a well-known biocontrol agent and plant growth promoter [10], further supports the beneficial impact of the BF.

A notable finding was that *Bacillus* species, which were a key component of the microbial inoculum, were not significantly enriched in the mature rhizosphere at the end of the experiment (8 months). However, this lack of late-stage persistence does not preclude their critical function in achieving disease suppression. The primary antagonistic role of *Bacillus* (e.g., producing antifungal lipopeptides) may have occurred during the secondary fermentation of the BF, effectively pre-conditioning the substrate [29]. Furthermore, *Bacillus* is well-known for its capacity for rapid, early-stage root colonization and as a potent trigger of Induced Systemic Resistance [30,44]. It is plausible that the *Bacillus* inoculum acted as a foundational catalyst in the early stages of plant growth, priming the plant’s defenses (as evidenced by the elevated POD and CAT activities) and facilitating the subsequent recruitment and proliferation of the taxa that dominated the mature rhizosphere, namely *Pseudomonas* and *Meyerozyma*.

Interestingly, the BF treatment led to divergent effects on bacterial and fungal diversity (Figure 4). The observed increase in bacterial diversity (Shannon index) is generally considered beneficial for soil health and ecosystem stability [9]. In contrast, the fungal diversity decreased, as indicated by the significantly lower Simpson’s index. This was driven by the strong dominance of *Meyerozyma*. This apparent paradox, i.e., increased bacterial diversity alongside decreased, specialized fungal diversity, highlights a potential mechanism of action. A more diverse bacterial community may provide broad, system-level benefits (e.g., enhanced nutrient cycling), while the targeted enrichment of a highly effective fungal antagonist (*Meyerozyma*) provides direct and potent disease suppression.

### 4.3. Functional Implications of Rhizosphere Microbiome Shifts

The predicted functional profiles of the microbial communities provided further insights. The significant reduction in the relative abundance of the ‘Endophyte-Plant Pathogen’ guild in the BF treatment (Figure 5A) strongly suggests a community-level functional shift away from pathogenic potential, which aligns perfectly with the observed disease suppression [53,54]. For bacteria, the upregulation of pathways related to Biotin metabolism, Amino sugar and nucleotide sugar metabolism, and RNA degradation, in the BF group (Figure 5B) points towards enhanced microbial metabolic activity and nutrient cycling [55,56]. Biotin is a crucial coenzyme, and its enhanced metabolism may reflect active bacterial growth [57]. Conversely, the downregulation of pathways related to exopolysaccharide biosynthesis and biofilm formation is notable. While a direct link to the fungal pathogen is speculative, this may reflect a shift in bacterial community strategies within the rhizosphere. However, as these functions are predicted from marker genes, these findings should be considered hypotheses that require confirmation through metatranscriptomic or metaproteomic analyses.

### 4.4. Linking Microbiome Changes to Plant Health and Disease Suppression

**Figure 7:**
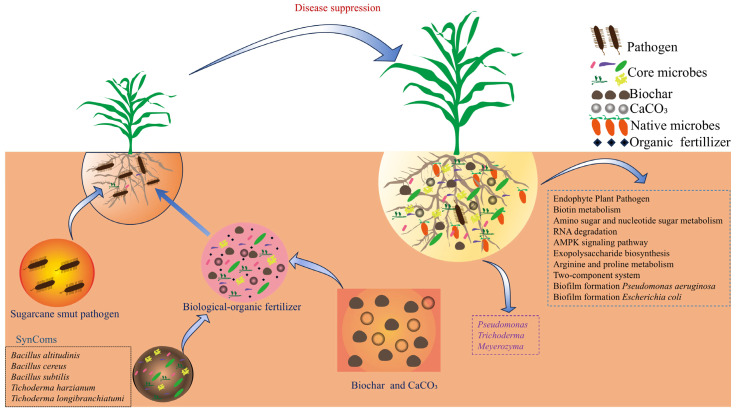
Schematic model showing the bio-organic fertilizer-mediated regulation of rhizosphere microbiome, plant growth, and suppression of sugarcane smut disease. The model illustrates how BF application, influences the sugarcane rhizosphere, which leads to the enrichment of core beneficial microbes (e.g., *Pseudomonas*, *Trichoderma*, *Meyerozyma*), alters soil microbial functions (e.g., increased beneficial metabolic pathways, decreased biofilm formation associated with pathogens), and physiological responses (e.g., increased antioxidant enzyme activity), and ultimately suppresses sugarcane smut and improves plant health.

The correlation and random forest analyses (Figure 6) strongly support the role of the enriched microbial taxa, particularly *Pseudomonas*, *Meyerozyma*, and *Trichoderma*, in the observed benefits. Their positive correlation with growth parameters and negative correlation with smut incidence highlight them as key players. Our proposed model (Figure 7) posits that the BF acts as a multi-functional soil conditioner and a microbial inoculum. It modifies the rhizosphere environment (e.g., higher pH and available K), leading to the recruitment and proliferation of a beneficial microbial consortium dominated by genera like *Pseudomonas* and *Meyerozyma*. This altered microbiome, in synergy with the direct effects of the BF components, contributes to improved soil nutrient availability (as shown by elevated AP and AK), enhanced plant physiological status (including antioxidant capacity), and suppression of pathogens like *S. scitamineum*, ultimately leading to superior growth and reduced disease.

### 4.5. Limitations and Future Directions

We acknowledge a limitation in our study design: the control fertilizer did not contain the amendments (biochar, CaCO_3_), which, along with the microbial inoculants, are integral components of the final BF formulation. This design tests the efficacy of the complete BF package against a conventional fertilizer, rather than isolating the specific effects of the microbial SynCom from the amendments. The observed benefits are thus the result of the synergistic interplay between the organic substrate, the amendments (which altered soil pH and K availability), and the microbial community. Future studies should include additional controls (e.g., compost + amendments but no microbes) to rigorously disentangle these complex interactions and pinpoint the relative contributions of each component.

## 5. Conclusions

This study provides compelling evidence that a novel bio-organic fertilizer system can significantly promote sugarcane growth and suppress smut disease. These beneficial effects are driven by the BF’s ability to synergistically modulate the rhizosphere environment and its microbial community. This modulation led to an enrichment of beneficial taxa such as *Pseudomonas* and *Meyerozyma*, an increase in bacterial diversity, and a functional shift in the fungal community, including a reduction in plant-pathogenic guilds, which further explains the observed outcomes. This work underscores the potential of engineering the soil microbiome through targeted, multi-component amendments as a strategy for sustainable sugarcane cultivation. Future research should focus on field-scale validation and further dissecting the molecular interactions between the key beneficial microbes, the sugarcane plant, and the smut pathogen using multi-omics approaches such as metagenomics (e.g., MAGs) and metatranscriptomics (RNA-seq).

## Figures and Tables

**Table 1:** Soil physicochemical properties under control and bio-organic fertilizer treatments. Values are means ± standard errors (n = 5). Different letters within a row indicate significant differences between the two treatments (independent samples *t*-test, *p* < 0.05).

Variables	Control	Bio-Organic Fertilizer
pH	6.82 ± 0.04 b	6.99 ± 0.04 a
Soil organic carbon (g/kg)	21.73 ± 2.88 a	21.46 ± 0.60 a
Total N (g/kg)	1.91 ± 0.02 a	2.08 ± 0.24 a
Total P (g/kg)	2.62 ± 0.33 a	2.38 ± 0.10 a
Total K (g/kg)	25.17 ± 3.02 a	20.33 ± 1.30 a
Available N (mg/kg)	200.1 ± 20.5 a	190.3 ± 18.7 a
Available P (mg/kg)	51.2 ± 0.9 a	63.4 ± 1.2 a
Available K (mg/kg)	880.5 ± 10.1 b	2130.7 ± 60.3 a

## Data Availability

The data (raw 16S rRNA sequencing data and ITS sequencing data) presented in this study are openly available in National Center for Bioinformation at https://ngdc.cncb.ac.cn/gsa, reference number CRA030229.
